# Supplementary material for: IHNV Infection Induces Strong Mucosal Immunity and Changes of Microbiota in Trout Intestine
Source: Viruses. 2022 Aug 22;14(8):1838. doi: 10.3390/v14081838 (PMC9415333; doi:10.3390/v14081838)
Supplement: Supplementary file 1 [file viruses-14-01838-s001.zip › viruses-1856450-supplementary.pdf]

viruses

MDPI

Table S1. Gene-specific primers used for quantitative real-time PCR in this study.

Gene	Gen Bank Accession No.	Primer Sequence (5′-3′)	
		Forward Primer	Reverse Primer
**IHNV-*G***	M16023.1	CACGGAAACAACACCACCATTA	AACAGCAAGGAGGAGAACAAGG
EF-1α	NM_001124339.1	CAACGATATCCGTCGTGGCA	ACAGCGAAACGACCAAGAGG
CCL19	AIN40032.1	GTTTCCCTCGCCACTTCAA	GCCACCCACTTGCTCTTTG
IL-8	NM_001124362.1	TGTCGTTGTGCTCCTGG	CCTGACCGCTCTTGCTC
IRF8	KT345962.1	CCGAGGAGGAGCAGAAGAGTAAAAG	GCGGCATTGAAAGAACCCAT
C1s	XM_021581979.1	TGAACAACCTGAACACCCC	CAGCCTATTAGCCTGTAACTCC
IRF1	NP_001239293.1	CGAGACTACACCAGACCCTA	TTGCTTTTGACCTCTTGTTATT
MHC I	XM_021579754.2	ACAGCTGTGAGTGGGTTGAG	GTCACAGGATAGGA
IgT	AY870265.1	TGTCCGTCCACATTCTC	TTGCCTTTCTTGGTTTT
pIgR	XM_021599266.1	TGTTACACTCCGCATTCTC	CAGGGCAGGTTTCTGATTT
MHC IIA	DQ246664.1	GGTGAGTTTGTTGGATAC	AGCGTTAGGCTTACATAGA
CD22	XP_014056970.1	TGAAGATGACAGTGGCAGAT	GGAGGGTTACAGGTGGAG
IgM	EF438413.1	CTATGGAATAGGCTGGAT	TGTTGCTGGAAGTGTCTC
IgD	AY870261.1	CATCCTGAGTGGGTTCT	GTAGGTGGTCCTTGTGC
STAT1	NP_001118179.1	CTCATCCCCTGGACCAAGTT	TTATTGTAGCCCTCCACCCA
IFNAR	AGO14285.1	CAGAGCCTCAGGAAGAACT	CAAGGGGTAGAAGAGCATA
MDA5	NP_001182108.1	CAGTGGAGATGACGATGGG	ACTTGGCGTTCTTGTGCTT
Vig1	NM_001124253.1	CAGGCCAGGTGAAGACTAGTAA	GCTGCAAAGATGCACTGGAG
IFNG	NM_001124620.1	GCTGTTCAACGGAAAACCTGTTT	TCACTGTCCTCAAACGTG
Mx1	XM_021567440.1	GATGCTGCACCTCAAGTCCTACTA	CGGATCACCATGGGAATCTGA
